# Sequence and expression analysis of rainbow trout CXCR2, CXCR3a and CXCR3b aids interpretation of lineage-specific conversion, loss and expansion of these receptors during vertebrate evolution

**DOI:** 10.1016/j.dci.2014.03.002

**Published:** 2014-08

**Authors:** Qiaoqing Xu, Ronggai Li, Milena M. Monte, Yousheng Jiang, Pin Nie, Jason W. Holland, Chris J. Secombes, Tiehui Wang

**Affiliations:** a Scottish Fish Immunology Research Centre, School of Biological Sciences, University of Aberdeen, Aberdeen AB24 2TZ, UK; b School of Animal Science, Yangtze University, Jingzhou, Hubei Province 434020, China; c College of Fishery and Life Science, Shanghai Ocean University, Shanghai 201306, China; d State Key Laboratory of Freshwater Ecology and Biotechnology, Institute of Hydrobiology, Chinese Academy of Sciences, Wuhan, Hubei Province 430072, China

**Keywords:** Rainbow trout, CXCR2, CXCR3a, CXCR3b, Evolution, Expression

## Abstract

The chemokine receptors CXCR1–3 bind to 11 chemokines (CXCL1–11) that are clustered on the same chromosome in mammals but are largely missing in ray-finned fish. A second CXCR1/2, and a CXCR3a and CXCR3b gene have been cloned in rainbow trout. Analysis of CXCR1–R3 genes in lobe-finned fish, ray-finned fish and tetrapod genomes revealed that the teleostomian ancestor likely possessed loci containing both CXCR1 and CXCR2, and CXCR3a and CXCR3b. Based on this synteny analysis the first trout CXCR1/2 gene was renamed CXCR1, and the new gene CXCR2. The CXCR1/R2 locus was shown to have further expanded in ray-finned fish. In relation to CXCR3, mammals appear to have lost CXCR3b and birds both CXCR3a and CXCR3b during evolution. Trout CXCR1–R3 have distinct tissue expression patterns and are differentially modulated by PAMPs, proinflammatory cytokines and infections. They are highly expressed in macrophages and neutrophils, with CXCR1 and CXCR2 also expressed in B-cells.

## Highlights

• The cDNA sequences of CXCR2, CXCR3a and CXCR3b have been cloned in rainbow trout.
• The linked CXCR1/CXCR2 and CXCR3a/CXCR3b loci are hypothesised to have been present in the teleostomian ancestor.
• CXCR1 and CXCR2 have likely undergone gene conversion whilst CXCR3b has been lost in mammals.
• Compared with mammals, ray-finned fish possess more CXCR1–R3 receptors, but fewer ligands.
• Trout CXCR1–R3 are expressed in macrophages and neutrophils, with CXCR1/R2 also abundant in B-cells.

## 1 Introduction

The coordinated movement of leucocytes is critical to both innate and adaptive immune systems and is mediated primarily by the chemokine system that includes a large number of ligands binding to a smaller number of receptors (Zlotnik and Yoshie, 2012; Bajoghli, 2013; Nomiyama et al., 2013). Mice and humans possess more than 40 chemokine ligands and 18 signalling chemokine receptors. The chemokine ligands have been classified into four groups (CC, CXC, CX3C, and XC) based on the position of the first two cysteine residues (Nomiyama et al., 2010; Alejo and Tafalla, 2011). Chemokine receptors are seven transmembrane molecules connected by three intracellular loops (ICL) and three extracellular loops (ECL), and have a conserved DRY amino acid motif within the second ICL, which is involved in coupling to G-proteins (Zlotnik and Yoshie, 2012). The chemokine receptors are classified according to the chemokine group that they bind, e.g. chemokine receptors binding CXC-chemokine ligands are referred to as CXCR followed by the appropriate number.

The analysis of vertebrate genomes (DeVries et al., 2006; Nomiyama et al., 2010, 2011; Chen et al., 2013) has revealed that both chemokines and their receptors have been evolving rapidly through species-specific gene duplications, although this type of rapid evolution is more characteristic of the chemokines than their receptors. Thus, the repertoire of chemokines differs between species, even within the same lineage. For example, human and mouse genomes contain 44 and 38 chemokines, respectively (Zlotnik and Yoshie, 2012), with an even greater variability observed in the teleost lineage, where zebrafish, medaka, stickleback and tetraodon contain 89, 36, 24 and 20 chemokines, respectively (Nomiyama et al., 2013). There are 17 CXC ligands described in mammals, with mice lacking CXCL8, and humans lacking CXCL15. CXCL1–11 are clustered on human chromosome (CH) 4, with CXCL13 further downstream. Of note, the clustered CXCL1–11 chemokines bind to three signalling receptors, namely CXCR1–3. CXCR1 binds only to CXCL6 and CXCL8, CXCR2 binds to CXCL1–3 and CXCL5–8, whilst CXCR3 binds to CXCL4 and CXCL9–11. CXCR1 and CXCR2 are key regulators of acute inflammatory responses by mediating neutrophil infiltration into inflammatory sites and activation of neutrophil functions (Coelho et al., 2008). In addition to their roles as mediators of inflammation, both receptors have been shown to play important roles in angiogenesis, haematopoiesis and cancer (Verbeke et al., 2011). CXCR3 is a chemokine receptor that is highly expressed in effector T cells and plays an important role in T cell trafficking and function (Groom and Luster, 2011).

The potential chemokine ligands binding to CXCR1–R3 are largely missing in ray-finned fish, with only three types of CXCL8 homologues (potentially able to bind to CXCR1 or CXCR2) and two types of CXCL11 homologues (potentially able to bind to CXCR3) found in some fish species (Chen et al., 2013). However, CXCR1/R2 like genes have been described from several fish species including common carp *Cyprinus carpio* (Fujiki et al., 1999), rainbow trout *Oncorhynchus mykiss* (Zhang et al., 2002), fugu *Fugu rubripes* (Saha et al., 2007), mandarin fish *Siniperca chuatsi* (Chen et al., 2009) zebrafish *Danio rerio* (Oehlers et al., 2010) and miiuy croaker *Miichthys miiuy* (Xu et al., 2013). CXCR3 has also been reported in grass carp *Ctenopharyngodon idella* (Chang et al., 2007) and medaka *Oryzias latipes* (Aghaallaei et al., 2010). Furthermore, multiple CXCR1–3 loci have been predicted in a few teleosts for which their genome sequences are available (DeVries et al., 2006; Nomiyama et al., 2010, 2011). Up to three CXCR1/R2 related loci and even more CXCR3-related loci are present in zebrafish, medaka and tetraodon (Nomiyama et al., 2011). Despite these findings, the orthologous relationship of fish CXCR1–3 to mammalian counterparts is obscure, in contrast to CXCR4 and CXCR5, also known in teleost fish, where the relationships are clear. For example, the origin of the teleost CXCR1/R2 loci was considered different from that of mammalian CXCR1/R2, which are highly identical in the same mammalian species and it had been hypothesized they were generated by gene duplication early in the amniote lineage (Nomiyama et al., 2013). Thus, a better understanding of the phylogenetic relationships of these receptors is an important prerequisite to understanding the evolution of chemokine ligand–receptor interactions in the context of host-pathogen responses.

Despite the presence of sequence data in the databases, functional analysis of CXCR1–R3 is scarce, especially in economically important fish species such as rainbow trout. A gene with homology to mammalian CXCR1 and CXCR2 (IL-8R-like) has been previously reported in rainbow trout (Zhang et al., 2002). In this study, we cloned a second trout gene with homology to CXCR1/CXCR2 and two CXCR3 genes (termed CXCR3a and CXCR3b). Phylogenetic tree and synteny analysis of lobe-finned fish, ray-finned fish (including the new trout molecules) and tetrapod molecules suggests that the ancestors of fish and tetrapods had loci containing CXCR1 and CXCR2, and CXCR3a and CXCR3b in their genomes. These receptor genes have experienced lineage-specific conversion, loss, and expansion in teleosts and tetrapods. We have analysed the expression of these receptors, *in vivo*, in a range of tissues from healthy trout, in kidney tissue during bacterial and parasitic infection and, *in vitro*, in stimulated primary head kidney macrophages, purified neutrophils and B cells.

## 2 Materials and methods

### 2.1 Fish

Rainbow trout were purchased from the Mill of Elrich Trout Fishery (Aberdeenshire, UK) and maintained in 1-m-diameter aerated fibreglass tanks supplied with a continuous flow of recirculating freshwater at 14 ± 1 °C. Fish were fed twice daily on standard commercial pellets (EWOS), and were acclimated for at least 2 weeks prior to experimentation.

### 2.2 Cloning and sequence analysis of CXCR2, CXCR3a and CXCR3b in rainbow trout

Blast (the basic local alignment search tool, Altschul et al., 1997) search was performed at NCBI (http://blast.ncbi.nlm.nih.gov/Blast.cgi) using mammalian CXCR2 and CXCR3 protein sequences, resulting in the identification of candidate ESTs for salmonid CXCR2 (acc. nos. CX355704, DW566408, EV375674 and EV378656) and two CXCR3 genes (acc. nos. CA343700 and CA381081). Primers (Table 1) were designed within the 5′-untranslated region (UTR) of each EST and used for 3′-RACE as described previously (Wang and Secombes, 2003; Wang et al., 2008), using 3′-RACE-ready cDNA samples prepared from head kidney (HK) for CXCR2 and RTS-11 cells for CXCR3a and CXCR3b (Ganassin and Bols, 1998). Cloning, sequencing and protein sequence analysis was performed as described previously (Wang et al., 2011a; Hong et al., 2013). Programs used included: ClustalW (Chenna et al., 2003) for multiple sequence alignments, MatGAT program (V2.02, Campanella et al., 2003) for global sequence comparisons, SMART7 (Letunic et al., 2012) for transmembrane domain prediction, MEGA5.2 (Tamura et al., 2011) for phylogenetic tree analysis and Genomicus (Muffato et al., 2010) for synteny analysis.

### 2.3 Comparative expression analysis of CXCR1, CXCR2, CXCR3a and CXCR3b

#### 2.3.1 Real-time PCR analysis of gene expression

Primer design, quality control and real-time RT-PCR analysis were performed as described previously (Wang et al., 2011a,b; Hong et al., 2013). A common reference containing an equi-molar amount of purified PCR products representing the four trout chemokine receptors was used for quantification. Primers used for real-time PCR detection are detailed in Table 1.

#### 2.3.2 Tissue distribution of gene expression

Six healthy rainbow trout (mean + SEM = 142 + 9 g) were anaesthetised, killed and seventeen tissues including; blood, HK, caudal kidney, spleen, thymus, gills, intestine, adipose tissue, brain, heart, muscle, ovary, liver, scales, skin, adipose fin, and tail fins, were sampled. The RNA preparation and RT-PCR analysis was performed as described previously (Hong et al., 2013). In all cDNA samples, the expression of each gene was calculated relative to the expression level of the house keeping gene, elongation factor (EF)-1α.

#### 2.3.3 Modulation of the expression of chemokine receptors in primary macrophages

Primary HK macrophage cultures were prepared from four individual fish, as outlined by Costa et al. (2011). At day 4 primary macrophages were stimulated with polyinosinic:polycytidylic acid (polyI:C, 50 μg/ml, Sigma), peptidoglycan (PGN, 5 μg/ml, Invivogen), trout recombinant (r) IL-1β (20 ng/ml, Hong et al., 2001), rIFN-γ (20 ng/mL, Wang et al., 2011b), rIL-6 (100 ng/mL, Costa et al., 2011) and rTNF-α3 (10 ng/mL, Hong et al., 2013) for 2, 4, 8 and 24 h. RNA extraction and real-time PCR analysis was conducted as described above.

### 2.4 Expression of chemokine receptors during bacterial infection

A pathogenic strain (MT3072) of the Gram-negative salmonid pathogen, *Yersinia ruckeri*, was injected intraperitoneally (i.p.) (1 × 10^6^ cfu in 0.5 ml PBS), as described previously (Wang et al., 2009; Harun et al., 2011). A control group was injected with PBS only (0.5 ml/fish). Six fish were killed at 6, 24, 48 and 72 h post-infection and spleen tissue collected for total RNA extraction. Real-time PCR analysis was conducted as described earlier.

### 2.5 Expression of chemokine receptors during parasitic infection

Proliferative kidney disease (PKD) is a parasitic disease of salmonid fish caused by the myxozoan parasite *Tetracapsuloides bryosalmonae*. The parasite infects salmonid fish primarily via the gill and skin epithelia, subsequently gaining access to internal tissues via the vascular system with the kidney being the main target organ for further development. Trunk kidney tissue collection and cDNA preparation was as described previously (Wang et al., 2010; Gorgoglione et al., 2013). The severity of clinical pathology was analysed and a kidney swelling index assigned to each fish according to the kidney swelling index system devised by Clifton-Hadley and colleagues (Clifton-Hadley et al., 1987). Real-time PCR analysis was conducted as described above.

### 2.6 Expression of chemokine receptors in purified neutrophils, B-cells and primary macrophages

HK cells and primary HK macrophages were prepared as described previously (Costa et al., 2011; Wang et al., 2011a).

#### 2.6.1 Purification of B cells

Blood was collected from 4 rainbow trout and diluted in cell culture medium (CM, Leibovitz L-15 medium containing 10% foetal calf serum (FCS), 100 u/ml penicillin and 100 μg/ml streptomycin), and heparin (10 u/ml). Peripheral blood leucocytes (PBL) were collected from a 51% Percoll (Sigma–Aldrich) gradient and washed once in CM. Cells were adjusted to 1 × 10^7^ cells/ml in IF medium (PBS supplemented with 2% FCS and 0.05% sodium azide; Sigma–Aldrich) and B cells were purified by magnetic-activated cell sorting (MACS) using an anti-trout IgM (I-14) monoclonal antibody (DeLuca et al., 1983) and anti-mouse IgG1 beads (Miltenyi Biotec). Purified B cells were >90% IgM positive as assessed by flow cytometry, and were dissolved in TRI Reagent (Sigma–Aldrich) for subsequent RNA extraction.

#### 2.6.2 Purification of neutrophils

Head kidney leucocytes were collected from four fish and suspended in CM supplemented with heparin (10 u/ml). Cells were fractionated using a 34–51% discontinuous Percoll gradient (at 400 g for 30 min). The granulocyte-enriched fraction at the 34–51% interface was retrieved and washed twice in IF medium. Neutrophils were purified by magnetic cell isolation (MACS) using the anti-trout neutrophil monoclonal antibody 5E9 (kindly provided by Dr. Chihaya Nakayasu, Aquatic Animal Health Division, National Research institute of Aquaculture, Japan) (Sasaki et al., 2002). Purified neutrophils, assessed to be >80% 5E9 positive by flow cytometry were dissolved in TRI Reagent for subsequent RNA extraction.

### 2.7 Statistical analysis

Real-time PCR data were analysed using the SPSS Statistics package 19.0 (SPSS Inc., Chicago, Illinois), as described previously (Wang et al., 2011a; Hong et al., 2013). One way-analysis of variance (ANOVA) and the LSD post hoc test were used to analyse the expression data in Figs. 8–10, with *p* < 0.05 between treatment groups and control groups considered significant. Since the *in vitro* expression data consisted of sets of samples from individual fish, a paired-sample *T*-test was applied (Figs. 6 and 7).

## 3 Results

### 3.1 Sequence analysis of trout CXCR2, CXCR3a and CXCR3b

The cloning and analysis of cDNA sequences for the three chemokine receptors (CXCR2, CXCR3a and CXCR3b) in rainbow trout are detailed in supplemental Figs. S1–S3 and summarised in Table 2. Each cDNA sequence possesses an in-frame stop codon upstream of the main open reading frame (ORF) and a polyadenylation signal before the poly A tail in the 3′ untranslated region (UTR), confirming the presence of the complete ORF and 3′ UTR. The CXCR3b gene possesses a large 3′-UTR of 2065 bp containing four ATTTA motifs, which may be indicative of post-transcriptional regulation (Khabar, 2010). All four receptors have a seven transmembrane (TM) domain structure and a putative N-glycosylation site in the first extracellular domain (Supplemental Figs. S1–S3).

A CXCR1/2-like gene has been isolated in rainbow trout previously by Zhang et al. (2002). This gene shared similar aa sequence identities to mammalian CXCR1 and CXCR2 (32.4–37.4% identities), but higher identity (53.5%) to carp CXCR1, the first CXCR1/2-like gene identified in fish (Fujiki et al., 1999). In the light of synteny analysis (described later), we have re-named the CXCR1/2-like gene isolated by Zhang et al. (2002) as trout CXCR1 and the new CXCR1/2-like gene as trout CXCR2. At least two CXCR1/2 related genes were identifiable in several model fish species with known genome sequences (Nomiyama et al., 2011). CXCR1 and CXCR2 in tetrapods exhibit high sequence identities (e.g. 82.7% in lizard and 75.8% in human) whereas, in contrast, teleost CXCR1 and CXCR2 molecules, within a species, share relatively low aa identity (33.1–43.3%) (Table 3). Although teleost CXCR1 and CXCR2 share high aa identities in different fish species, both molecules have similar aa identities to CXCR1 and CXCR2 in tetrapod species. In general, teleost CXCR2 share a higher aa identity to both tetrapod CXCR1 and CXCR2 (Table 3).

**Fig. 1:**
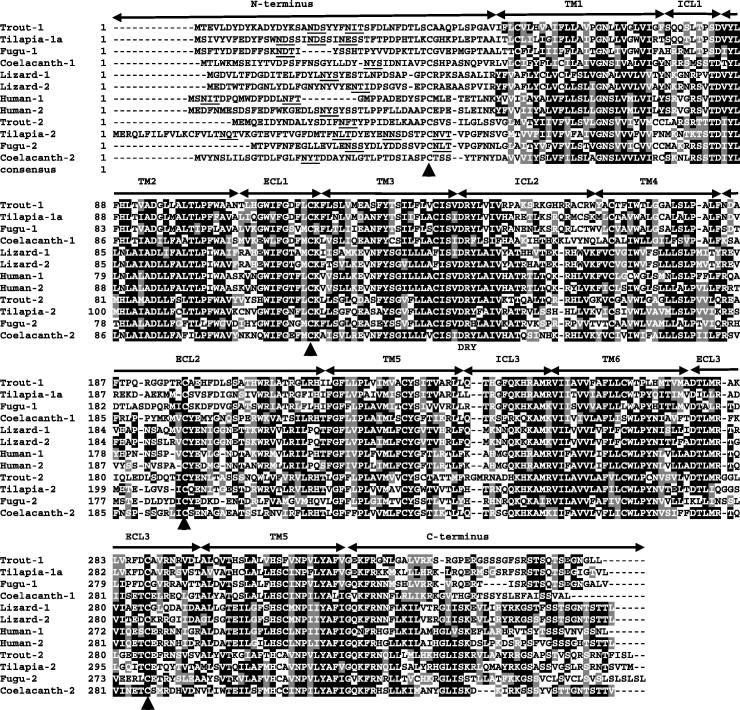
Multiple alignments of vertebrate CXCR1 and CXCR2. The multiple alignment was produced using ClustalW, and conserved amino acids shaded using BOXSHADE (version 3.21) except in the N-terminal domain. The N-terminus, seven transmembrane domains (TM1–7), three extracellular loops (ECL1–3) and intracellular loops (ICL1–3) and the C-terminus are marked above the alignment. The four conserved cysteine residues in each extracellular domain are indicated by black arrows and the DRY motifs below the alignment. Potential N-glycosylation sites in the N-terminal domain are underlined. The accession numbers for sequences used in this alignment are given in Fig. 3.

The structure of trout CXCR2 was predicted to have seven-transmembrane domains, with an extracellular N-terminus and an intracellular C-terminus. Due to the high aa identities of teleost CXCR2 to mammalian CXCR1 and CXCR2, a multiple alignment was constructed from CXCR1 and CXCR2 molecules from selected mammalian (human), reptile (lizard), teleost (ray-finned) fish species known to have experienced a further whole genome duplication (WGD) event (trout, fugu and tilapia *Oreochromis niloticus*), and the lobe-finned coelacanth *Latimeria chalumnae* (Fig. 1). Vertebrate CXCR1 and CXCR2 are well conserved in the seven TM domains with sequences all possessing four cysteine residues (one in the N-terminal and three in the extracellular loops; ECLs) that form two disulphide bonds, one from the N-terminal to ECL3 and one from ECL1 to ECL2 (Limatola et al., 2005), and the DRY motif in ICL2. There are also 1–3 potential N-glycosylation sites present in the N-terminus of both CXCR1 and CXCR2, with the exception of lizard CXCR2. Sequence alignments suggest that fish CXCR2 molecules are more closely related to tetrapod CXCR1 and CXCR2 as illustrated by the amino acid deletion in ICL2 and the amino acid insertion in TM4 relative to fish CXCR1 (Fig. 1).

**Fig. 2:**
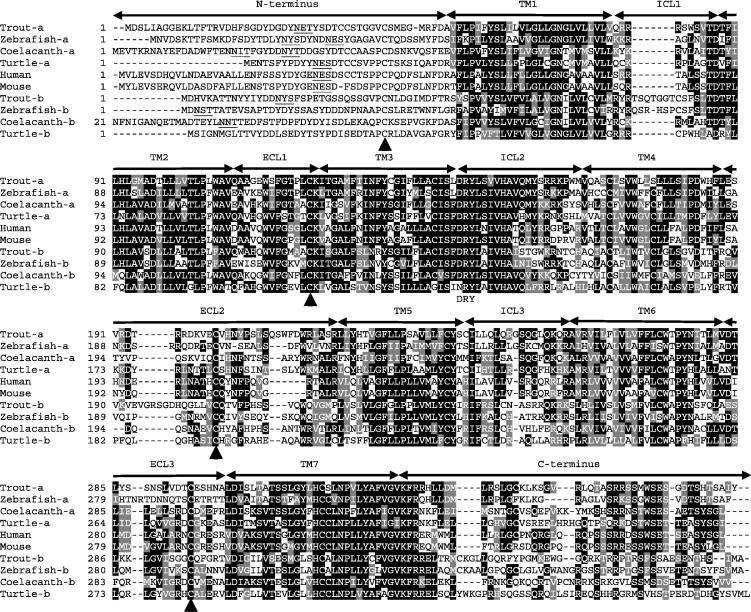
Multiple alignments of CXCR3a, CXCR3b and mammalian CXCR3. The multiple alignment was produced using ClustalW, and conserved amino acids shaded using BOXSHADE (version 3.21) except in the N-terminal domain. The N-terminus, seven transmembrane domains (TM1–7), three extracellular loops (ECL1–3) and intracellular loops (ICL1–3) and the C-terminus are marked above the alignment. The four conserved cysteine residues in each extracellular domain are indicated by black arrows and the DRY motifs below the alignment. Potential N-glycosylation sites in the N-terminal domain are underlined. The accession numbers for sequences used in this alignment are given in Fig. 3.

One CXCR3 gene is present in mammals but none have been found in birds (Zlotnik and Yoshie, 2012). Surprisingly, we found two CXCR3-related genes in amphibians, reptiles and lobe-finned fish, and up to three in ray-finned fish in the ENSEMBL database. Trout CXCR3a and CXCR3b share only 36.9% aa identity (Table 4). In general, fish CXCR3a, along with CXCR3a from amphibians (frog) and reptiles (turtle) share high aa identities to each other (39.6–55.8%) and to mammalian CXCR3 molecules (41.5–49.7%), whilst exhibiting lower aa identities to CXCR3b molecules (32.4–43.3%). A multiple alignment of selected CXCR3a, CXCR3b, and mammalian CXCR3 molecules highlighted general conservation in the seven TM domains, the four cysteine residues in the extracellular domains, the DRY motif, and the potential N-glycosylation sites in the N-terminus (Fig. 2).

### 3.2 The common ancestor of fish and tetrapods had a locus containing CXCR1 and CXCR2 in its genome

**Fig. 3:**
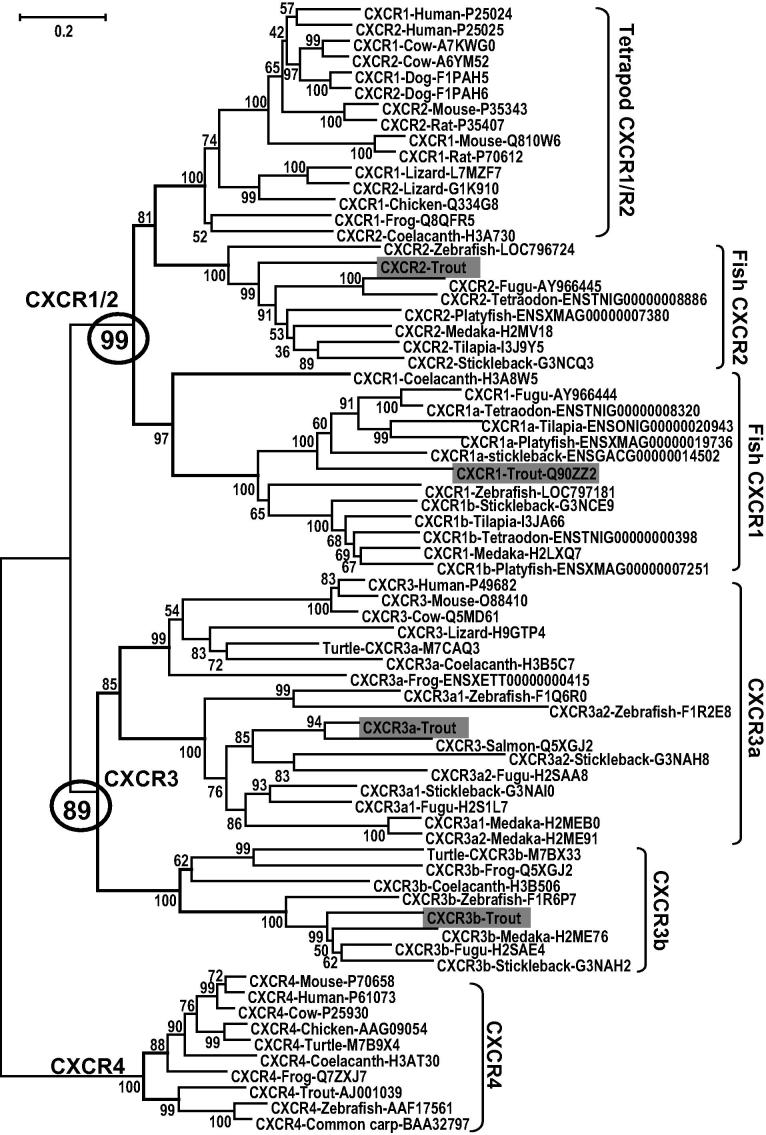
An unrooted phylogenetic tree of vertebrate CXCR1–4 molecules. The tree was constructed using amino acid multiple alignments and the neighbour-joining method within the MEGA5 program (Tamura et al., 2011). Node values represent percent bootstrap confidence derived from 10,000 replicates. The evolutionary distances were computed using the JTT matrix-based method. All positions containing alignment gaps and missing data were eliminated only in pairwise sequence comparisons. The accession number for each sequence is given after the molecular type and species name except for trout CXCR2, CXCR3a and CXCR3b that are provided in Table 2.

Fish CXCR1/R2 molecules have been described in a few fish species; however their relationships to mammalian CXCR1/2 are not clear. We first constructed a neighbour joining phylogenetic tree using CXCR1–4 molecules from representative ray-finned fish (zebrafish, medaka, tilapia, tetraodon, fugu and platyfish *Xiphophorus maculatus*), a lobe-finned fish (coelancanth) that is a close living relative of tetrapods (Amemiya et al., 2013), an amphibian (frog *Xenopus tropicalis*), reptiles (anol lizard or turtle *Pelodiscus sinensis*), a bird (chicken *Gallus gallus*) and a selection of mammals (human, cow, dog, mouse and rat) (Fig. 3). It was clear that all the vertebrate CXCR1 and CXCR2 molecules form a clade separate to the CXCR3 and CXCR4 clades. The CXCR1/R2 clade was further divided into three subclades. All the tetrapod CXCR1 and CXCR2 molecules, along with the lobe-finned fish CXCR2 formed one subclade, we have termed tetrapod CXCR1/R2 group. The fish CXCR2 molecules formed a second subgroup, whilst the fish CXCR1 molecules, with the lobe-finned fish CXCR1 at the root of the group, formed the third subgroup. It is noteworthy that the ray-finned fish CXCR1 were divided into CXCR1a and CXCR1b subgroups.

Phylogenetic analysis alone does not give a clear explanation as to how the fish and tetrapod CXCR1 and CXCR2 molecules may have originated. For example, two different evolutionary paths could be put forward. In one the teleost CXCR1 and CXCR2 genes could have been generated by the fish-wide WGD and the mammalian CXCR1 and CXCR2 genes by a gene duplication early in the amniote lineage, as suggested by Nomiyama et al. (2011, 2013). However, the presence of CXCR1 and CXCR2 clades in different teleost fish and the coelacanth may indicate an alternative evolutionary pathway based on the premise that the teleostomian ancestor already possessed the CXCR1 and CXCR2 genes that were preserved in lobe-finned fish, converted in reptiles and mammals, and duplicated in ray-finned fish.

**Fig. 4:**
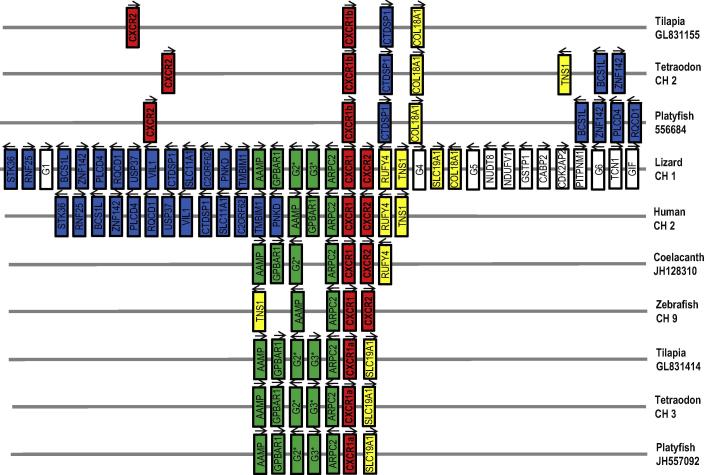
Diagram to show gene synteny at the CXCR1/CXCR2 loci in vertebrates. The green anole lizard CXCR1/CXCR2 locus was used as a reference to compare the conserved synteny between the CXCR1/R2 loci in lobe-finned fish (Coelacanth), ray-finned fish (zebrafish, tilapia, platyfish and tetraodon), reptiles (lizard) and mammals (humans). The arrows indicate the transcriptional direction. G1 = ENSACAG00000002813, G2 = ENSACAG00000001282, G3 = ENSACAG00000001171, G4 = ENSACAG00000025725, G5 = ENSACAG00000025482 and G6 = ENSACAG00000009990.

To help clarify the evolutionary relationships of these molecules, synteny analysis was carried out using the Genomicus database v73.01 using the lizard CXCR1/R2 locus as a reference. Both human and lizard CXCR1 and CXCR2 are syntenically well conserved, as evidenced by large blocks of conserved genes on lizard CH 1 and human CH 2 (Fig. 4). The CXCR1/CXCR2 loci of both the lobe-finned coelacanth and ray-finned zebrafish are also syntenically conserved (e.g. the presence of AAMP and ARPC2 genes close to CXCR1 and CXCR2), which may represent the ancestral state. Two syntenically conserved chromosomal loci containing CXCR1/R2 genes were found in at least three model fish species (tilapia, tetraodon and platyfish) (Fig. 4), suggesting that these loci may have been generated by the teleost-wide WGD event. Together the synteny and the phylogenetic analysis provides evidence that the CXCR1 and CXCR2 genes were present in the ancestor of fish and tetrapods and were duplicated in the ray-finned fish via the WGD, with differential evolution of the loci in teleost fish and tetrapods that will be discussed later.

### 3.3 Two CXCR3 genes (a and b) existed in the teleostomian ancestor

**Fig. 5:**
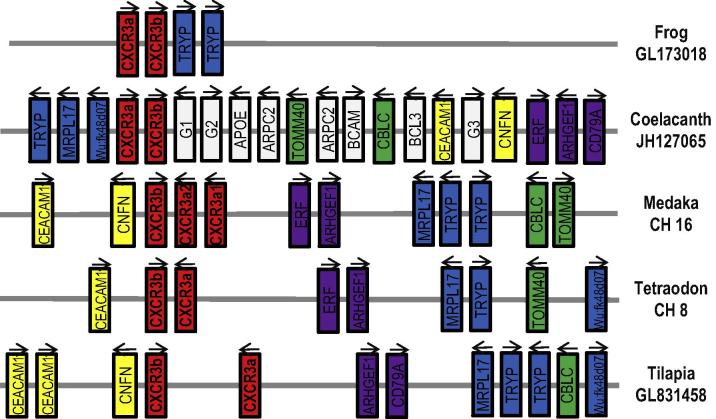
Diagram to show gene synteny at the CXCR3a/CXCR3b loci in fish and amphibians. The lobe-finned fish coelacanth CXCR3a/CXCR3b locus was used as a reference to compare the conserved synteny between the CXC3a/CXCR3b in ray-finned fish (tilapia, tetraodon and medaka), and in amphibians (frog). The arrows indicate the transcriptional direction. G1 = ENSLACG00000014632; G2 = ENSLACG00000014632 and G3 = ENSLACG00000010635.

Phylogenetic analysis (Fig. 3) suggests that there are two types of CXCR3, CXCR3a and CXCR3b, present in fish, amphibians and reptiles, with only a single CXCR3 molecule equivalent to the CXCR3a type in mammals and no CXCR3 molecules found in birds (Nomiyama et al., 2013). CXCR3a and CXCR3b genes in amphibians, lobe-finned and ray-finned fish are located together on the same chromosome and show conserved gene synteny (Fig. 5). Some ray-finned fish possess more CXCR3 paralogues (e.g. medaka possesses one CXCR3b and two CXCR3a genes) generated by local gene duplication events). However, only poor gene synteny was found between the fish and mammalian CXCR3 loci. Nevertheless, fish and amphibian synteny, together with the phylogenetic tree analysis, support the premise that the CXCR3a and CXCR3b genes were present in the teleostomian ancestor, and were preserved in lobe-finned fish, ray-finned fish, amphibians and potentially in reptiles. CXCR3b was apparently lost in mammals, whereas both isoforms have been lost in birds.

### 3.4 Tissue distribution of the expression of different chemokine receptors

**Fig. 6:**
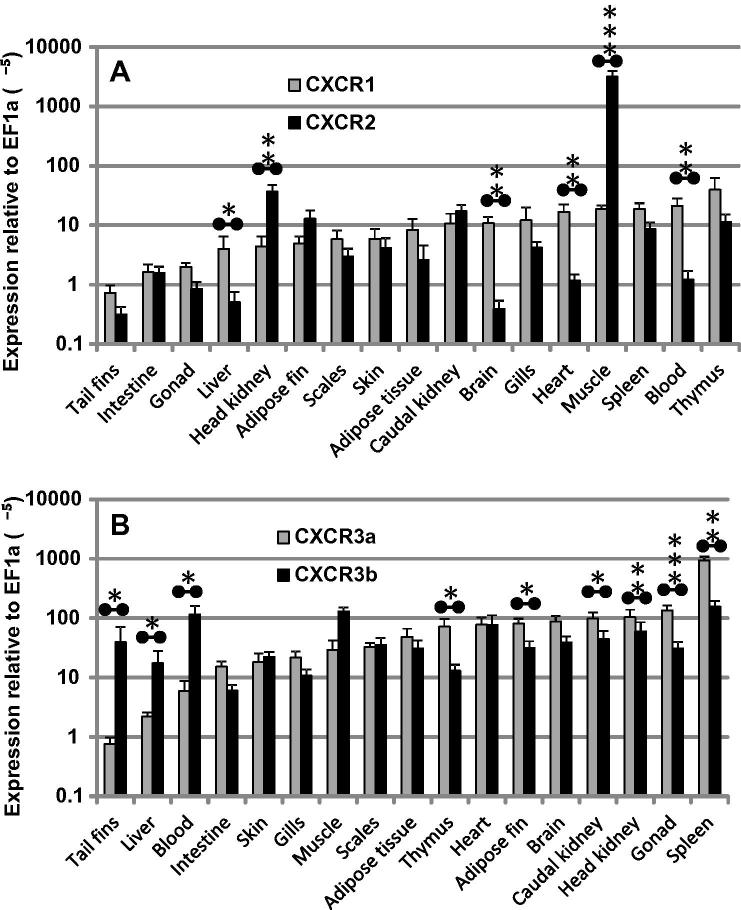
Tissue distribution of expression of chemokine receptors. The expression of trout CXCR1, CXCR2, CXCR3a and CXCR3b in seventeen tissues from six healthy fish was determined by real-time PCR. Transcript levels of chemokine receptors are presented relative to that of EF-1α. The means + SEM are shown. The expression levels of CXCR1 and CXCR2, and CXCR3a and CXCR3b, in each tissue were compared by paired sample *T*-tests, and the *p* values are shown above the bars when there is significant difference. ^∗^*p* ⩽ 0.05, ^∗∗^*p* ⩽ 0.01 and ^∗∗∗^*p* ⩽ 0.001.

The relative expression levels of trout CXCR1, CXCR2, CXCR3a and CXCR3b were examined in seventeen tissues from six healthy fish by real-time PCR (Fig. 6). The expression of all the receptors was detectable in all tissues examined. High constitutive expression of CXCR1 was present in immune-relevant tissues, e.g. thymus, blood, spleen and gills, as well as in non-immune tissues such as muscle, heart, brain and caudal kidney. The expression of CXCR2 was particularly high in muscle, and relatively high in HK, caudal kidney and thymus. The expression of CXCR1 was significantly higher than CXCR2 in liver, brain, heart and blood, whilst CXCR2 was significantly higher than CXCR1 in HK and muscle. Interestingly, the relative expression levels of CXCR1 and CXCR2 did not differ in many immune relevant tissues such as thymus, spleen, gills, intestine and skin (Fig. 6A).

The expression of CXCR3a and CXCR3b was generally higher compared to that of CXCR1 and CXCR2, with CXCR3b expression showing relatively little variation between tissues (Fig. 6). The highest expression level of CXCR3a was observed in spleen tissue, whilst CXCR3b was highly expressed in spleen, muscle and blood. CXCR3a expression was significantly higher than CXCR3b in thymus, adipose fin, caudal kidney, head kidney, gonad, and spleen. CXCR3b expression was significantly higher than CXCR3a in tail fins, liver and blood (Fig. 6B).

### 3.5 Differential modulation of chemokine receptors in primary HK macrophages

**Fig. 7:**
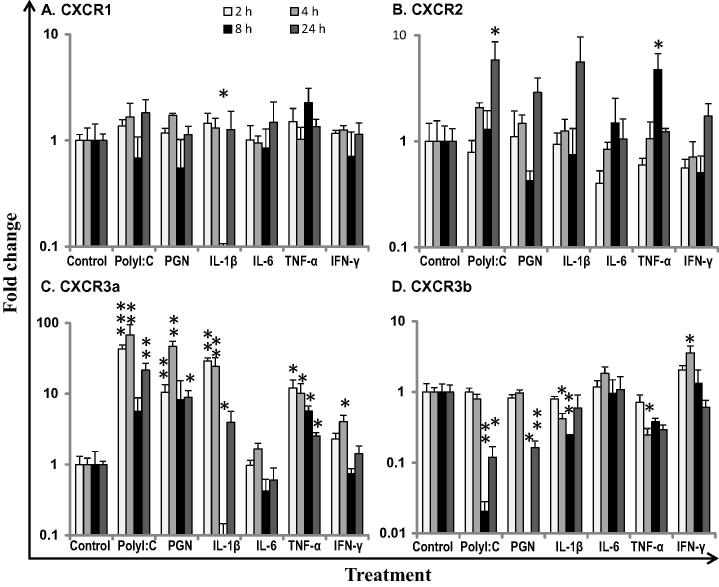
Modulated expression of trout chemokine receptors in primary HK macrophages. Four day old primary HK macrophages were stimulated with PAMPs (polyI:C and PGN), and recombinant trout cytokines (IL-1β, IL-6, TNF-α and IFN-γ) for 2, 4, 8 and 24 h. Quantification of gene expression was as described in Fig. 6. Modulated expression was expressed as a fold change calculated as the mean expression levels in stimulated cells normalized to that of time-matched controls. The means + SEM of cells from four fish are shown. *p*-values generated by paired sample *T* tests between stimulated and time-matched control samples are shown above the bars. ^∗^*p* ⩽ 0.05, ^∗∗^*p* ⩽ 0.01 and ^∗∗∗^*p* ⩽ 0.001.

**Fig. 8:**
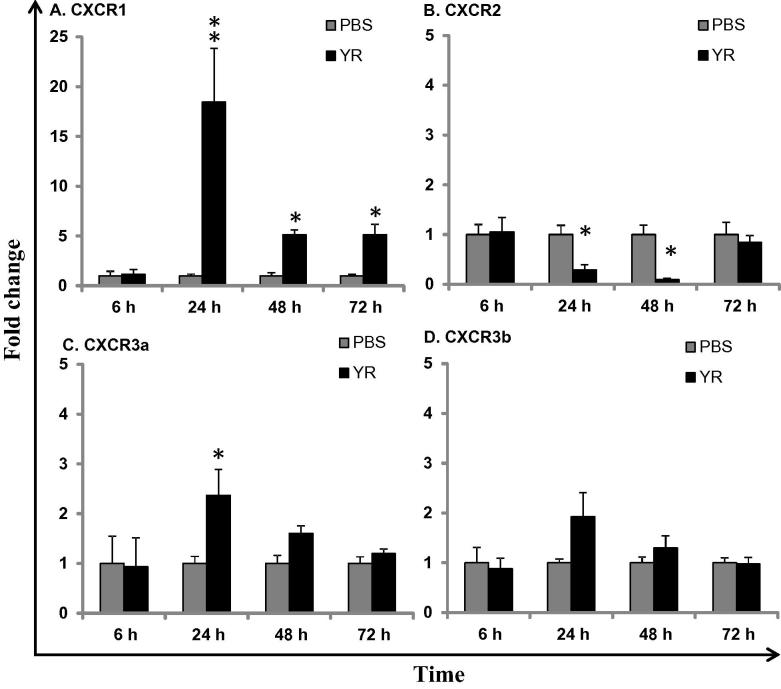
Modulation of trout chemokine receptor expression by bacterial infection. Rainbow trout were i.p. injected with *Y. ruckeri* (YR) or PBS as control. HK tissue was collected at 6, 24, 48 and 72 h post-challenge and real-time PCR analysis performed as described in Fig. 6. Results, presented as a fold change relative to the controls, are means + SEM of five fish. The significance of LSD post hoc tests after one way-analysis of variance between infected and control fish is shown above the bars. ^**∗**^*p* ⩽ 0.05 and ^∗∗^*p* ⩽ 0.01.

Mammalian CXCR1 and CXCR2 (Morohashi et al., 1995) and fish CXCR3 (Wang et al., 2008; Aghaallaei et al., 2010) are known to be expressed in monocytes/macrophages. We, therefore, examined the modulation of chemokine receptor expression in primary HK macrophages stimulated by PAMPs and proinflammatory cytokines (Fig. 7). All four chemokine receptors were highly expressed in trout primary HK macrophages. CXCR1 expression remained refractory to all stimulants, the only exception being the IL-1β-mediated down-regulation of CXCR1 expression at 8 h (Fig. 7A). CXCR2 expression was moderately induced by polyI:C at 24 h and by TNF-α at 8 h but was refractory to PGN, IL-1β, IL-6 and IFN-γ stimulation (Fig. 7B). Both CXCR3a and CXCR3b were moderately up-regulated by IFN-γ (up to 4-fold) at 4 h post-stimulation but were refractory to IL-6. Interestingly, the expression of CXCR3a was markedly up-regulated by polyI:C (up to 68-fold), PGN (up to 47-fold), IL-1β (up to 29-fold) and TNF-α (up to 12-fold) (Fig. 7C). In sharp contrast, the expression of CXCR3b was inhibited under the same stimulatory conditions (Fig. 7D).

### 3.6 Modulation of chemokine receptors by bacterial and parasitic infections

We further investigated chemokine receptor transcriptional modulation by bacterial and parasitic infections. Following infection of trout with a common Gram-negative bacterial pathogen, *Y. ruckeri*, the expression level of CXCR1 in HK was significantly up-regulated (up to 18.4-fold) from 24 to 72 h relative to time matched control fish. In contrast, CXCR2 expression was decreased at 24 and 48 h post-infection. A modest, yet significant increase of CXCR3a expression (2.4-fold) was observed at 24 h. CXCR3b expression remained refractory to bacterial infection.

**Fig. 9:**
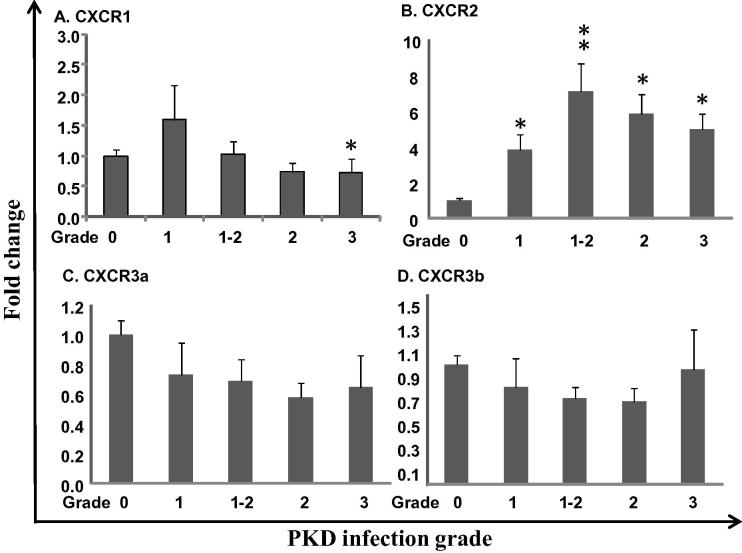
Modulation of trout chemokine receptor expression by parasite infection. Kidneys from rainbow trout infected with *T.bryosalmonae* (grade 1, 1–2, 2 and 3) or from uninfected (control, grade 0) fish were collected during a natural infection (Wang et al., 2010; Gorgoglione et al., 2013). Results, presented as a fold change relative to the control fish, are means + SEM. The numbers of fish analysed were 11, 5, 9, 10 and 9 representing control, Grade 1, 1–2, 2, and 3, respectively. The significance of LSD post hoc tests after one way-analysis of variance between infected and control fish is shown above the bars. ^**∗**^*p* ⩽ 0.05 and ^∗∗^*p* ⩽ 0.01.

To gain further insights into the immune mechanisms underlying the characteristic chronic lymphoid-driven immunopathology seen in *T. bryosalmonae* infected rainbow trout (Gorgoglione et al., 2013), the expression profiles of the chemokine receptors were investigated in fish with clinical PKD relative to uninfected controls. The expression of CXCR3a and CXCR3b was refractory to PKD (Fig. 9C and D), whilst CXCR1 decreased at advanced clinical stages (grade 3) (Fig. 9A). In contrast, CXCR2 expression was significantly up regulated in kidney tissue samples from grade 1 to grade 3 (Fig. 9B).

### 3.7 Expression of chemokine receptors in purified neutrophils and B cells

**Fig. 10:**
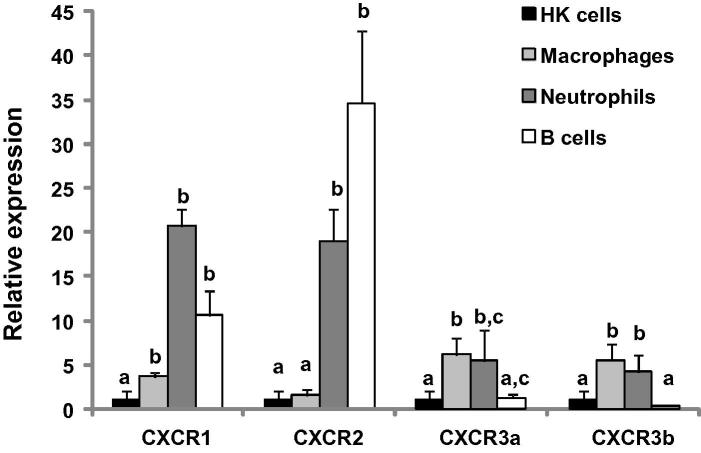
Relative expression of trout chemokine receptors in purified primary macrophages, neutrophils and B cells. HK leucocytes (Wang et al., 2011) and 4 day old primary macrophages (Costa et al., 2011) were prepared as described previously. Neutrophils and B cells were prepared by MACS using monoclonal antibodies 5E9 (Sasaki et al., 2002) and I-14 (DeLuca et al., 1983), respectively. The gene expression levels in purified cells for each receptor are presented relative to HK leucocytes in which the expression level was defined as 1. The means + SEM of four fish are shown. The expression levels between different cell populations are statistically significant (*p* < 0.05) where letters over the bars are different for each receptor.

To gain further insights into the cell types expressing these chemokine receptors, we investigated their expression in purified primary HK macrophages, neutrophils, and B cells using HK leucocytes as a reference, since all of the selected cell populations are present in HK leucocyte preparations. The primary HK macrophages highly express macrophage/dendritic cell markers such as MCSFR (Wang et al., 2008) and LAMP3/CD208 (Johansson et al., 2012). Purified neutrophils were >80% 5E9 positive (Sasaki et al., 2002) and B cells >90% IgM (I-14) positive (DeLuca et al., 1983). The expression of both CXCR1 and CXCR2 was significantly higher in MACS purified neutrophils and B cells compared to HK leucocytes. CXCR1 expression was also significantly higher in primary HK macrophages (Fig. 10). The expression of both CXCR3a and CXCR3b was higher in primary macrophages and neutrophils compared to HK leucocytes (Fig. 10).

## 4 Discussion

### 4.1 Evolution of CXCR1–3

#### 4.1.1 The ancestral state of fish and tetrapod CXCR1–R3

The high sequence identity between CXCR1 and CXCR2 in the same mammalian species and their linkage to the same chromosome suggests that the mammalian CXCR1 and CXCR2 genes have arisen by gene duplication early in the amniote lineage. The presence of (at least) two CXCR1/2 genes in teleosts has been proposed to be due to chromosomal duplication or the teleost fish-wide WGD (Nomiyama et al., 2013). However, our synteny and phylogenetic tree analysis, that included the lobe-finned coelacanth, suggests that two CXCR1/R2 like genes were already present in the ancestor of fish and tetrapods. Ray-finned fish appear to have expanded the CXCR1/R2 paralogues via the teleost-wide WGD event leading to three (CXCR1a, CXCR1b and CXCR2) genes in two genomic loci, as seen in three teleost fish species in this study. The presence of CXCR1 and CXCR2 as gene neighbours in both the lobe-finned coelancanth and ray-finned zebrafish suggests that this was the primordial state of the CXCR1 and CXCR2 locus that appears to have been preserved in reptiles and mammals, although with only one gene remaining in amphibians and birds. The possibility that CXCR1 and CXCR2 arose from a single CXCR1/R2 gene by gene duplication independently in both the mammalian and reptile lineages is less likely, as this process would have needed two independent events to have taken place: Firstly, the loss of one of the CXCR1 or CXCR2 genes followed by two gene duplication events. The high identity of CXCR1 and CXCR2 in mammals and reptiles may have resulted from gene conversion (Shields, 2000), driven by ligand/receptor co-evolutionary processes (discussed later).

In contrast to the common scenario where multiple related teleost genes reside in different chromosomal loci that have been generated by the teleost-wide WGD event (Wang et al., 2008; Husain et al., 2012; Hong et al., 2013), two or more CXCR3-related genes are found in close proximity to each other in several fish species, implying that they have arisen from local gene duplication events. The presence of clustered CXCR3a and CXCR3b genes in both lobe-finned and ray-finned fish, as well as in amphibians and potentially in reptiles may suggest that both genes were present in the genome of the teleostomian ancestor. However, mammals appear to have lost CXCR3b and birds both genes during vertebrate evolution. Importantly, birds have lost the known ligands of CXCR3 (i.e. CXCL9–11), which could account for the absence of CXCR3 in birds (Nomiyama et al., 2013).

#### 4.1.2 Coevolution of chemokines and their receptors

To maintain functional interactions, chemokine ligands and their receptors must both co-evolve so that changes in one partner at the points of interaction are complemented by changes in the other. Mammalian CXCR1 and CXCR2 bind to a cluster of proinflammatory CXC chemokines that seem to have originated from a lineage specific expansion in mammals. With the exception of CXCL8, most of these chemokines have not been found in birds, amphibians or fish (Nomiyama et al., 2013). Thus, mammalian CXCR2 binds to CXCL1–3 and CXCL5–8, whilst CXCR1 binds to CXCL6 and CXCL8, with the only exception known to date being the absence of CXCL8 in rodents (mouse and rat). The binding of multiple common ligands might be a driving force for the evolutionary conversion of mammalian CXCR1 and CXCR2. Potentially the selective pressure of CXCR1 and CXCR2 gene conversion in rodents has been relaxed owing to the absence of CXCL8. Indeed, rodent CXCR1 and CXCR2 are more divergent (58.7% aa identity) than in other mammals (e.g. 75.8% aa identity in human) and appear to be phylogenetically distinct, whereas in other mammalian species they group together. Evidence supporting CXCR1 and CXCR2 gene conversion being specific to non-rodent mammalian species has been obtained (Shields, 2000).

#### 4.1.3 Implication of more CXCR1–3 genes in fish

In humans, CXCR1–R3 bind to twelve CXC ligands (CXCL1–11 and CXCL4L1) (Nomiyama et al., 2010, 2013). According to the mammalian ligand-receptor binding paradigm, the five or more receptors (CXCR1a, CXCR1b, CXCR2, CXCR3a and CXCR3b) present in most ray-finned fish could bind up to five ligands (CXCL8_L1–3 and CXCL11_L1–2, Chen et al., 2013). Although little is known about the specific ligand-receptor pairing, the presence of more receptors suggests that more specific ligand-receptor pairings could exist in fish. Recently, CXCR2 was reported to be the functional receptor for CXCL8_L1 in zebrafish where it is known to be involved in neutrophil recruitment during local infections, even though both CXCR1 and CXCR2 are expressed in neutrophils (Deng et al., 2013). As to whether zebrafish CXCR1 is specific for other CXCL8-related molecules or both CXCR1 and CXCR2 bind to fish-specific CXC ligands remains to be determined. Nevertheless, the specific CXCL8_L1-CXCR2 pairing in zebrafish provides evidence supporting the existence of novel fish-specific chemokine-receptor pairings.

Of the four receptors discussed, CXCR3b is absent in mammalian genomes, whilst both CXCR3a and CXCR3b are absent in birds, the latter being consistent with the lack of binding ligands (CXCL9–11) in bird genomes. Two potential ligands, CXCL11_L1–2, have been described in several fish species (Chen et al., 2013). It will be interesting to see in future studies whether the two CXCR3 receptors bind to both CXCL11 ligands or if fish CXCR3b may partner with novel fish specific ligands, such as the CXCL-F ligands (Chen et al., 2013).

### 4.2 Functional implications of the gene expression analysis

Rainbow trout CXCR1, CXCR2, CXCR3a and CXCR3b are differentially expressed in different tissues from healthy fish. In general, CXCR1 and CXCR3b expression are less variable across different tissues, which may be indicative of a homeostatic role for these receptors. The high expression levels of CXCR1, CXCR2 and CXCR3a in immune relevant tissues, such as thymus, spleen, gills and HK, and differential modulation by PAMPs, cytokines, and pathogens, suggests that they are involved in immune regulation. Mammalian CXCR1 and CXCR2 are key regulators of inflammation and activators of neutrophils and CXCR3 is highly expressed in effector T cells. Whether this paradigm holds true in teleost fish warrants further investigation considering the difference of the ligands of CXCR1–R3 and the receptors between fish and mammals. To our surprise the highest expression levels of CXCR2 were observed in muscle, which was over two orders of magnitude higher than in immune tissues such as thymus, spleen and gill tissues, which may indicate a potential role of this receptor in the fish musculoskeletal system. Human CXCR2 is also expressed in skeletal muscle and is influenced by acute exercise at both the mRNA and protein levels and has been implicated in exercise-stimulated angiogenesis (Frydelund-Larsen et al., 2007). Thus, in fish, the high level of CXCR2 expression in fish muscle may relate to swimming behaviour.

An important prerequisite towards understanding the functional significance of CXCR1–R3 in fish is to determine the cell types that express them. Thus, we examined the expression of CXCR1–R3 in purified primary macrophages, neutrophils, and B cells. Even though our cell preparations did not reach 100% purity, the comparison of CXCR gene expression between the different cell types and to unfractionated HK leucocytes indicated that CXCR1 and CXCR2 are highly expressed in neutrophils and B cells, with CXCR1 also expressed abundantly in macrophages relative to total HK cells. Whilst it remains possible that gene expression in purified neutrophils and B cells was influenced by the MACS procedure, it is, nevertheless, clear that these cell types can express these receptors at relatively high levels. CXCR3a and CXCR3b were also abundantly expressed in macrophages and neutrophils. These data are consistent with CXCR3 studies in medaka where CXCR3 expression, *in vivo*, was found to be associated with myeloid markers and genes linked to antigen uptake and presentation and with grass carp studies describing the abundant expression of CXCR3 in macrophages and granulocytes (Chang et al., 2007; Aghaallaei et al., 2010). In mammals, CXCR3 is highly expressed in IL-2 activated T cells, but undetectable in resting T cells, B cells, monocytes and granulocytes. The exclusive expression of CXCR3 in activated T cells suggests that it may have a role in the selective recruitment of lymphocyte subsets (Groom and Luster, 2011). Our initial analysis on trout HK leucocytes revealed that expression of CXCR3a but not CXCR3b increased in response to stimulation with the T cell mitogen phytohemagglutinin (un-published data). However, the expression of CXCR3a and CXCR3b in fish T cells remains to be determined.

Macrophages produce proinflammatory cytokines (e.g. IL-1β, TNF-α and IL-6) in response to PAMPs (polyI:C and PGN). These proinflammatory cytokines, together with IFN-γ can then act to regulate macrophage function via different signalling pathways. In addition, the proinflammatoy cytokines can regulate each other, e.g. IL-1β upregulates the expression of TNF-α, and vice versa (Hong et al., 2001, 2013). The differential modulation of CXCR1–R3 expression in primary HK macrophages suggests that different pathways regulate the expression of these receptors. CXCR3a is upregulated rapidly by both PAMPs (polyI:C and PGN) and proinflammatoy cytokines (IL-1β and TNF-α), hinting that some common pathways of the TLR signalling and proinflammatory cytokine signalling may be involved in regulation of this receptor. The converse regulation of trout CXCR3a and CXCR3b by PAMPs and proinflammatory cytokines is particularly interesting. According to known mammalian CXC ligand receptor binding, only one potential CXCR3 ligand (γIP or CXCL11_L1), has been identified in rainbow trout to date (Laing et al., 2002a; Chen et al., 2013). γIP and CXCR3a are highly up regulated by polyI:C, IL-1β and TNF-α whilst, in contrast, CXCR3b expression was down-regulated. This may indicate that CXCR3a-γIP is a possible ligand-receptor pair in rainbow trout. Whether CXCR3a and CXCR3b bind to the same ligand or CXCR3b binds to other ligands remains to be determined.

### 4.3 CXCR1–3 in disease models

The expression of chemokine receptors has been examined in several fish disease models (Huising et al., 2003; Gonzalez et al., 2007; Forlenza et al., 2008). In this study, the expression of trout CXCR1–3 in response to an acute Gram-negative bacterial (*Y. ruckeri*) infection and a chronic parasitic (*T. bryosalmonae*) infection was investigated. Two distinct patterns of CXCR1 and CXCR2 expression were observed. Whilst the bacterial infection increased CXCR1 expression but decreased CXCR2 expression, PKD enhanced CXCR2 expression at all stages of clinical disease. It is not clear whether such changes in gene expression are the result of direct activation or inhibition of transcription, or due to the movement of cells that express the receptors. Apparently the cells expressing CXCR1 and CXCR2 can overlap. For example, both receptors are expressed in neutrophils and B cells, although this may not be the case with all cell types. Future studies aiming to identify cell populations expressing CXCR1 and CXCR2 in fish disease models may help to clarify the molecular mechanisms underpinning pathogen-mediated pathology.

Interestingly, the expression of IL-8 (CXCL8_L1, Chen et al., 2013) was up-regulated by *Y. ruckeri* infection (Raida et al., 2011), but refractory to *T. bryosalmonae* (Gorgoglione et al., 2013). So far, only one CXCL8 like molecule has been identified in rainbow trout (Laing et al., 2002b), even though up to three may exist in other fish species. The concomitant increase in IL-8 and CXCR1 expression in the bacterial infection model suggests that this is a likely chemokine ligand-receptor pairing with a functional role in this disease. The increased expression of CXCR2 but lack of IL-8 up-regulation in PKD may be indicative of additional chemokines, yet to be discovered in rainbow trout that could bind to CXCR2 and play a functional role in PKD pathology.

## 5 Conclusions

The analysis of CXCR1–R3 genes in lobe-finned, ray-finned fish and tetrapod genomes has revealed that the teleostomian ancestor likely possessed clustered CXCR1/CXCR2 and CXCR3a/CXCR3b loci. During vertebrate evolution, CXCR1 and CXCR2 appear to have undergone gene conversion in mammals probably driven by ligand/receptor co-evolutionary processes. Mammals appear to have lost CXCR3b, whilst birds have lost both CXCR3a and CXCR3b, along with their putative ligands. The CXCR1/R2 locus has been further expanded in ray-finned fish via the fish-wide WGD event. Thus, more CXCR1–R3 orthologues are present in this lineage despite fewer potential ligands being found to date. This implies that unique ligand-receptor pairings could exist in fish. Trout CXCR1–R3 have distinct tissue expression patterns and are differentially modulated by PAMPs and proinflammatory cytokines in primary head kidney macrophages and by bacterial and parasitic infection *in vivo*. All the receptors were found to be expressed in macrophages and neutrophils with CXCR1/R2 also highly expressed in B-cells.

## Figures and Tables

**Table 1:** Primers used for cloning and expression analysis.

Gene	Primer	Sequences (5′- to 3′)	Application
CXCR2	F1	CTGAGACTCC GAGACAGACACTCC	3′-RACE
	F2	CTGTCAAGTG GACATGTAAAGCCAG	3′-RACE
	F	GGACATGTAAAGCCAGCTCATGG	Real-time PCR
	R	AGGGTCAGGGAGAAGAGGAGGTC	Real-time PCR
CXCR3a	F1	CCCATCATCTCTGTGGAAACTGA	3′-RACE
	F2	CTGATTGACAGACTGCATCAATACC	3′-RACE
	F	CAAGGCAACCACAAATTACTATATTTATGATG	Real-time PCR
	R	CCCTCACAGACTCCAGGAAGTG	Real-time PCR
CXCR3b	F1	CGAGAAGAGTGTCCTGAGTC	3′-RACE
	F2	CTGTGAAGGTGTTTCAGGTGTTC	3′-RACE
	F	CACTGGAGCCATGTTTACAATCAACT	Real-time PCR
	R	CAGCACACACAGCACCAGGAT	Real-time PCR
EF-1a	F	CAAGGATATCCGTCGTGGCA	Real-time PCR
	R	ACAGCGAAACGACCAAGAGG	Real-time PCR

**Table 2:** Summary of sequence analysis of rainbow trout CXCR2, CXCR3a and CXCR3b.

Features	CXCR2	CXCR3a	CXCR3b
GenBank ID	HG794530	AJ888881	AJ888878
cDNA Length (bp)	1393	1541	3308
In frame stop codon^a^	Yes	Yes	Yes
ORF (bp)	1089	1125	1143
ORF (aa)	362	374	380
3′-UTR (bp)^b^	248	343	2065
5′ UTR (bp)	28	54	79
polyA signal	Yes	Yes	Yes
ATTTA motifs	0	0	4
N-glycoslyation sites	2	1	1

*Notes*.

a In frame stop codon before the main ORF in the 5′-UTR.

b The length of 3′-UTR excluding the poly A tail.

**Table 3:** Comparison of identities (top right) and similarities (bottom left) between CXCR1 and CXCR2 molecules from selected teleosts and tetrapods, and coelacanth. The accession numbers of the sequences used are detailed in Fig. 3.

	1	2	3	4	5	6	7	8	9	10	11	12	13	14	15
1. CXCR1-Trout		40.5	58.1	50.3	37.5	55.5	36.3	42.0	38.6	35.8	37.1	39.8	39.5	39.3	38.4
2. CXCR2-Trout	57.2		36.3	37.2	55.4	37.6	50.5	37.5	45.0	43.5	44.3	46.5	45.7	42.6	43.9
3. CXCR1a-Tilapia	72.1	58.3		44.2	34.4	62.2	31.6	40.1	34.3	33.9	33.7	37.6	38.0	36.9	38.1
4. CXCR1b-Tilapia	65.6	58.6	65.0		36.8	44.9	37.0	42.2	41.4	39.0	39.7	41.4	44.4	39.5	41.1
5. CXCR2-Tilapia	51.7	71.4	52.8	55.4		36.4	50.7	36.2	44.7	42.3	43.5	41.1	42.1	41.3	42.4
6. CXCR1-Fugu	70.8	56.1	77.4	66.7	52.3		33.1	40.6	35.1	35.2	36.3	35.5	36.0	39.0	35.6
7. CXCR2-Fugu	53.2	68.0	53.5	58.6	66.3	52.1		35.4	39.6	38.1	39.9	37.9	41.1	37.5	41.7
8. CXCR1-Coelacanth	60.7	59.7	60.2	63.3	57.6	59.5	56.9		43.3	42.4	42.0	42.7	41.9	39.8	44.2
9. CXCR2-Coelacanth	57.1	64.6	56.5	58.9	63.4	54.3	58.5	62.2		53.7	54.0	53.3	54.9	48.3	53.7
10. CXCR1-Lizard	54.6	65.5	56.5	59.7	59.7	56.0	58.5	62.4	71.0		82.7	56.5	57.4	49.2	55.8
11. CXCR2-Lizard	54.9	64.9	55.2	59.7	58.4	54.9	59.3	62.7	69.9	88.9		58.2	57.1	51.4	56.3
12. CXCR1-Human	56.0	65.5	57.4	59.7	58.4	56.0	56.6	61.8	70.9	73.5	72.4		75.8	64.7	67.1
13. CXCR2-Human	59.2	67.1	57.5	62.8	61.3	56.7	58.1	60.6	74.2	74.4	74.2	84.4		59.5	71.1
14. CXCR1-Mouse	55.4	62.4	57.1	57.8	59.9	58.5	55.7	62.3	65.0	64.1	65.5	76.6	71.9		58.7
15. CXCR2-Mouse	56.0	67.1	56.0	58.3	61.3	55.4	60.2	62.1	71.0	72.1	70.5	78.6	85.0	70.5	

**Table 4:** Comparison of identities (top right) and similarities (bottom left) of CXCR3 molecules from selected teleosts, tetrapods, and coelacanth. The accession numbers of the sequences used are detailed in Fig. 3 except for frog CXCR3a (a full-length protein sequence from *Xenopus laevis* with acc. no. K7ZRA2).

	1	2	3	4	5	6	7	8	9	10	11	12	13	14
1. Trout-a		55.8	37.6	46.1	41.3	46.2	45.5	42.3	45.3	36.9	37.9	38.5	36.0	37.1
2. Zebrafish-a1	73.5		38.5	42.7	39.6	40.9	41.5	41.8	42.0	33.2	36.9	35.0	33.5	32.4
3. Zebrafish-a2	58.0	56.7		30.1	29.2	32.0	30.8	29.5	30.8	26.4	26.4	28.2	27.3	28.5
4. Coelacanth-a	66.0	62.8	52.9		46.8	54.9	45.9	44.7	45.9	35.0	33.7	43.3	32.9	34.5
5. Frog-a	61.8	59.4	51.5	66.0		46.4	43.4	42.0	44.8	33.4	32.7	39.5	34.6	35.6
6. Turtle-a	64.2	62.4	50.8	71.7	65.8		49.7	48.4	49.6	35.4	33.8	40.1	37.3	38.1
7. Human	64.7	61.1	50.3	65.2	61.5	64.9		86.4	87.0	39.1	37.3	38.3	35.5	39.1
8. Mouse	61.0	61.9	49.7	62.6	62.9	64.0	91.8		84.5	37.9	36.2	38.4	35.1	37.6
9. Cow	63.9	62.9	49.5	64.7	62.9	65.6	92.4	89.9		38.4	35.4	40.4	35.9	38.1
10. Trout-b	58.2	52.4	47.6	57.9	53.7	54.7	55.8	55.8	53.7		57.8	40.9	39.3	40.9
11. Zebrafish-b	57.9	56.3	45.0	58.5	57.1	55.8	54.2	55.0	54.2	73.7		39.0	38.3	41.1
12. Coelacanth-b	55.2	54.7	48.4	58.2	57.0	56.2	55.4	55.2	56.2	59.7	61.3		41.3	41.6
13. Frog-b	55.9	52.3	45.5	54.8	55.2	57.6	53.0	55.3	53.0	57.1	55.8	57.7		47.6
14. Turtle-b	54.8	54.6	50.3	56.1	56.2	57.3	54.8	56.2	54.6	59.2	59.8	61.3	61.8	

## Appendix A Supplementary data

Supplementary Fig. S1 Nucleotide and deduced amino acid sequences of rainbow trout CXCR2 cDNA (EMBL accession number HG794530). The start and stop codons for translation, an in-frame start and stop codons in the 5′-UTR, and a polyadenylation signal in the 3′-UTR are highlighted in red. Potential N-glycosylation sites are in bold and underlined. The seven transmembrane domains are highlighted in green

Supplementary Fig. S2 Nucleotide and deduced amino acid sequences of rainbow trout CXCR3a cDNA (EMBL accession number AJ888881). The start and stop codons for translation, an in-frame stop codon in the 5′-UTR, and a polyadenylation signal in the 3′-UTR are highlighted in red. A potential N-glycosylation site is in bold underlined. The seven transmembrane domains are highlighted in green

Supplementary Fig. S3 Nucleotide and deduced amino acid sequences of rainbow trout CXCR3b cDNA (EMBL accession number AJ888878). The start and stop codons for translation, an in-frame stop codon in the 5′-UTR, and a polyadenylation signal in the 3′-UTR are highlighted in red. Potential N-glycosylation sites are in bold and underlined. Putative mRNA instability motifs (ATTTA) are in bold and boxed. The seven transmembrane domains are highlighted in green.
