# Supplementary material for: Sequence and expression analysis of rainbow trout CXCR2, CXCR3a and CXCR3b aids interpretation of lineage-specific conversion, loss and expansion of these receptors during vertebrate evolution
Source: Dev Comp Immunol. 2014 Aug;45(2):201–13. doi: 10.1016/j.dci.2014.03.002 (PMC4052464; doi:10.1016/j.dci.2014.03.002)
Supplement: Supplementary Fig. S1 — Nucleotide and deduced amino acid sequences of rainbow trout CXCR2 cDNA (EMBL accession number HG794530). The start and stop codons for translation, an in-frame start and stop codons in the 5′-UTR, and a polyadenylation signal in the 3′-UTR are highlighted in red. Potential N-glycosylation sites are in bold and underlined. The seven transmembrane domains are highlighted in green [file mmc1.docx]

1 M E M Q E I D Y N D A L Y S D I F **N F T** Y

2 TGTCAAGTGGACATGTAAAGCCAGCTCATGGAAATGCAAGAAATTGACTATAATGATGCACTCTACTCCGACATCTTCAACTTCACCTAT

22 P P I D E L K A A P C S V S I L G L S S V G L M V T Y I I V

92 CCTCCCATAGACGAGCTCAAGGCAGCCCCCTGTAGTGTGTCTATCTTGGGCTTGAGCAGTGTTGGTCTGATGGTCACATACATCATTGTG

52 F V L S V L G N S V V I Y V M C C L A R S R T T T D I Y L M

182 TTTGTCCTAAGTGTGCTGGGCAACAGTGTGGTCATCTACGTGATGTGCTGCTTGGCCAGGAGCCGGACCACCACAGACATCTACCTGATG

82 H L A M A D L L F S L T L P F W A V Y V Y S H W I F G T F L

272 CACCTAGCCATGGCCGACCTCCTCTTCTCCCTGACCCTCCCCTTCTGGGCCGTCTACGTCTACTCTCACTGGATCTTTGGTACCTTCCTC

112 C K L L S G L Q D A S F Y S G V F L L A C I S V D R Y L A I

362 TGTAAGCTCCTGTCTGGCCTCCAGGATGCTTCCTTTTATAGTGGGGTCTTCCTGTTAGCGTGCATTAGCGTGGACCGCTACCTGGCTATC

142 V K T T Q A L T Q R R H L V G K V C G A V W L G A G L L S L

452 GTGAAGACCACGCAGGCGCTGACTCAACGTCGCCACCTGGTGGGGAAAGTTTGTGGAGCCGTGTGGCTGGGGGCAGGGCTTCTCTCATTG

172 P V V L Q R E A I Q L E D L S D Q T I C Y E **N L T** A S S S N

542 CCTGTGGTGCTCCAGCGGGAAGCTATCCAACTGGAGGATCTCAGCGACCAGACCATCTGCTACGAGAACCTGACTGCGTCAAGCAGCAAC

202 Q W L V F V R V L R H T L G F F L P L A V M V V C Y S C T A

632 CAGTGGCTGGTTTTTGTGCGGGTGCTTCGCCACACACTGGGATTCTTCCTGCCGCTGGCAGTCATGGTCGTCTGTTACAGCTGCACGGCG

232 T T M F R G M R N A D H K H K A M R V I L A V V L A F V L C

722 ACGACGATGTTCCGTGGCATGCGCAACGCCGACCATAAACACAAGGCCATGCGCGTCATCCTGGCCGTGGTGTTGGCATTCGTGTTATGT

262 W L P C N V S V L V D T L M R G G L L G E E T C E F R N S V

812 TGGCTGCCGTGCAATGTCAGCGTGCTGGTAGACACATTGATGCGAGGCGGCTTGCTGGGCGAGGAGACATGTGAGTTCCGGAACAGTGTG

292 S V A L Y V T K G I A F T H C A V N P V L Y A F I G Q K F R

902 AGTGTGGCGCTGTACGTGACCAAGGGGATAGCGTTCACGCACTGCGCAGTCAACCCCGTGCTGTACGCCTTCATCGGGCAGAAGTTCCGG

322 N Q L L L M L H K H G L I S K R V L A A Y R R G S A P S T V

992 AACCAGCTCCTGCTGATGCTCCACAAGCATGGGCTGATCAGCAAGAGGGTGCTGGCCGCTTACCGCAGGGGCTCGGCCCCCAGCACGGTC

356 S Q R S R N T F I S L

1082 AGTCAAAGGTCTAGGAACACCTTTATTAGCCTGTAAGGTTTCAGTAGTGAGGATTCAATATTTTGTCACATAGCTAGCTTTGCTTTTATT

1172 TGTTTTTGTTTTTGATGTGCATTGTCTTTTAGTATATTGCAACATGTTTGACACTATCCCATTAGTTTTTTTCCCATTATATTAATCAAA

1262 ATATAGTTTTTCATGTGATACTTTAATGCTTGCTAAGTATAAACATTAATGTCAAGGCCTAATCTGTACGAAATAAAACTGAATCTTCCA

1352 ACTTTTACTTGTGGAAAAAAAAAAAAAAAAAAAAAAAAAAAA

**Fig. S1. Nucleotide and deduced amino acid sequences of rainbow trout CXCR2 cDNA (EMBL accession number HG794530).** The start and stop codons for translation, an in-frame start and stop codons in the 5’-UTR, and a polyadenylation signal in the 3’-UTR are highlighted in red. Potential N-glycosylation sites are in bold and underlined. The seven transmembrane domains are highlighted in green.
