# Supplementary material for: Sequence and expression analysis of rainbow trout CXCR2, CXCR3a and CXCR3b aids interpretation of lineage-specific conversion, loss and expansion of these receptors during vertebrate evolution
Source: Dev Comp Immunol. 2014 Aug;45(2):201–13. doi: 10.1016/j.dci.2014.03.002 (PMC4052464; doi:10.1016/j.dci.2014.03.002)
Supplement: Supplementary Fig. S2 — Nucleotide and deduced amino acid sequences of rainbow trout CXCR3a cDNA (EMBL accession number AJ888881). The start and stop codons for translation, an in-frame stop codon in the 5′-UTR, and a polyadenylation signal in the 3′-UTR are highlighted in red. A potential N-glycosylation site is in bold underlined. The seven transmembrane domains are highlighted in green [file mmc2.docx]

1 M D S L I A G G E K L T

1 CCCATCATCTCTGTGGAAACTGATTGACAGACTGCATCAATACCATTCAAAGGCATGGATTCCCTCATTGCCGGCGGTGAAAAGTTAACA

13 F T R V D H F S G D Y D G D Y **N E T** Y S D T C C S T G G V C

91 TTTACCAGAGTTGACCACTTCTCTGGTGATTATGATGGGGATTATAACGAGACCTACAGTGACACGTGCTGTTCTACTGGGGGGGTGTGC

43 S M E G M R F D A V F L P I F Y S L I L V L G L L G N G L V

181 AGCATGGAGGGCATGCGTTTTGACGCTGTGTTCCTCCCCATATTCTACTCCCTGATATTGGTTCTTGGGCTGCTGGGCAACGGGCTGGTA

73 L L V L V Q R R R S W S V T D T F I L H L G M A D T L L L V

271 CTTTTGGTTCTGGTACAGAGGAGGCGGAGCTGGAGCGTGACTGACACCTTCATCCTGCACCTGGGCATGGCTGACACCCTGCTGCTGGTC

103 T L P L W A V Q A A G E W S F G T P L C K I T G A M F T I N

361 ACGCTGCCCCTCTGGGCTGTTCAGGCCGCTGGGGAATGGAGCTTTGGGACACCCCTCTGCAAGATCACTGGAGCCATGTTTACAATCAAC

133 F Y C G I F L L A C I S L D R Y L S V V H A V Q M Y S R R K

451 TTTTACTGTGGCATCTTCCTGCTGGCCTGCATCAGTCTGGACCGCTACCTGTCCGTGGTCCACGCAGTCCAGATGTACTCTCGCAGGAAG

163 P W M V Q A S C L S V W L L S L L L S I P D W H F L E S V R

541 CCCTGGATGGTGCAGGCCAGCTGCCTGTCCGTGTGGCTCCTCTCCCTCCTCCTCTCCATCCCCGACTGGCACTTCCTGGAGTCTGTGAGG

293 D T R R D K V E C V H N Y P S L S Q S W F D W R L A S R L L

631 GACACCAGACGAGACAAAGTGGAGTGTGTTCACAACTACCCGTCCCTCTCCCAGTCTTGGTTTGACTGGCGCCTGGCCTCCCGCCTGCTC

223 Y H T V G F L L P S A V L L F C Y S C I L L Q L Q R G S Q G

721 TACCACACGGTGGGCTTCCTCCTCCCCTCTGCCGTGCTACTCTTCTGCTACTCCTGCATCCTGCTGCAGCTGCAGCGTGGCTCCCAGGGC

253 L Q K Q R A V R V I L F L V L V F F L C W T P Y N I T L M V

811 CTCCAGAAGCAGAGGGCCGTCCGGGTCATCCTGTTCCTGGTGCTGGTCTTCTTCCTCTGCTGGACGCCCTACAACATCACCCTTATGGTG

283 D T L Y S S N S L V D T C E S H N A L D I S L T A T S S L G

901 GACACCCTCTACTCCAGCAACTCCCTGGTGGACACCTGCGAGAGCCATAATGCCCTGGACATCTCCCTGACGGCCACCTCTTCTCTGGGC

313 Y L H C S L N P V L Y A F V G V K F R R H L L D M L R S L G

991 TACCTGCACTGCAGCCTCAACCCCGTGCTCTACGCCTTCGTGGGGGTGAAGTTCCGGCGCCACCTGCTGGACATGCTGAGGTCCCTGGGC

343 C K L K S G V R L Q T A S R R S S M W S E S G D T S H T S A

1081 TGCAAGCTGAAGAGTGGAGTCAGGCTGCAGACTGCCAGCCGGAGGAGTTCCATGTGGTCTGAGTCTGGAGACACCTCCCACACCTCTGCC

373 I Y

1171 ATCTATTAAGTACTTCTCTCTATCCCAAGATTTCCAAAACTGGGTCCTAAGGACCCCAAGGGGTGCATGTTTTGTTTTTTGCCCTAGCAC

1261 TACACATTTGATACAAATAATCAGCTAATCGTCAAGCTTTGATGATTTGAATCGGCTGTGTAGTGTTGGGGCGAAAACCCGGGGCGTGCA

1351 CCCCTTGGTGTCTGAGGACCGAGTTTAGGAAACGCTGCTCTACGCTCTATCCTGTTGCTCTTGAGTTGTCGTTGACTCAATAAGTGGATT

1441 ATCAATGTGACCTTTAGCCTACTCCAACTTCCAAATTATATTTCCATTATAAAAACAAATATTAAAAACATTTGAGTATAGCAAAAAAAA

1531 AAAAAAAAAAA

**Fig. S2. Nucleotide and deduced amino acid sequences of rainbow trout CXCR3a cDNA (EMBL accession number AJ888881).** The start and stop codons for translation, an in-frame stop codon in the 5’-UTR, and a polyadenylation signal in the 3’-UTR are highlighted in red. A potential *N*-glycosylation site is in bold underlined. The seven transmembrane domains are highlighted in green.
