# Supplementary material for: Sequence and expression analysis of rainbow trout CXCR2, CXCR3a and CXCR3b aids interpretation of lineage-specific conversion, loss and expansion of these receptors during vertebrate evolution
Source: Dev Comp Immunol. 2014 Aug;45(2):201–13. doi: 10.1016/j.dci.2014.03.002 (PMC4052464; doi:10.1016/j.dci.2014.03.002)
Supplement: Supplementary Fig. S3 — Nucleotide and deduced amino acid sequences of rainbow trout CXCR3b cDNA (EMBL accession number AJ888878). The start and stop codons for translation, an in-frame stop codon in the 5′-UTR, and a polyadenylation signal in the 3′-UTR are highlighted in red. Potential N-glycosylation sites are in bold and underlined. Putative mRNA instability motifs (ATTTA) are in bold and boxed. The seven transmembrane domains are highlighted in green. [file mmc3.docx]

1 M D H V

2 GAACGCGGGTGAGACTACGAGAAGAGTGTCCTGAGTCTGTGAAGGTGTTTCAGGTGTTCCGCTGTTTCGTTGGTAACAATGGATCACGTC

5 K A T T N Y Y I Y D D **N Y S** F S P E T G S S Q S S G V P C N

92 AAGGCAACCACAAATTACTATATTTATGATGACAACTACAGCTTTTCACCAGAAACAGGCAGTAGCCAATCCAGTGGCGTGCCCTGCAAC

35 L D G I M D F T R S Y S P V V Y S L V F V L A L V G N I L V

182 CTGGATGGCATCATGGACTTCACCCGGAGCTACTCCCCTGTGGTCTACAGCCTGGTGTTTGTGCTGGCGCTGGTGGGTAACATCCTGGTG

65 L C V L M R Y R T S Q T G G T C S F S L T D T F L L H L A V

272 CTGTGTGTGCTGATGCGCTACCGCACCTCTCAGACAGGTGGGACCTGCTCCTTCTCCCTCACCGACACCTTCCTGCTCCACCTGGCCGTG

95 S D L L L A L T L P L F A V Q W A H Q W V F G M A A C K I S

362 TCCGACCTCCTGCTGGCCCTCACGCTGCCCCTGTTCGCCGTCCAGTGGGCCCACCAGTGGGTGTTCGGCATGGCCGCCTGCAAGATTTCC

125 G A L F S L N R Y S G I L F L A C I S F D R Y L A I V H A I

452 GGAGCCCTGTTCTCTCTGAACCGCTACAGCGGCATCTTGTTCCTGGCCTGCATCAGCTTCGACCGCTACCTGGCCATTGTCCACGCCATC

155 S T G W K R N T C H A Q I A C T L I W T V C L G L S G V D I

542 AGCACCGGCTGGAAACGCAACACCTGCCATGCACAGATTGCCTGCACCCTGATCTGGACAGTGTGCTTGGGCCTGAGTGGGGTGGACATC

185 T F R Q V V K V E V G R S G D H Q G L L V C Q T V F P H S S

632 ACCTTTAGACAAGTGGTGAAGGTGGAAGTGGGGCGCTCGGGGGATCATCAGGGCCTGCTGGTGTGCCAGACGGTGTTCCCCCACAGCTCA

215 V Q W Q V G M P L V S L V L G F G L P P L V M L Y C Y I R I

722 GTGCAGTGGCAGGTGGGGATGCCACTAGTCAGCTTGGTGCTGGGTTTTGGGCTGCCCCCGCTGGTCATGCTCTACTGCTACATCCGTATC

245 F R S L C N A S R R Q K R K S L H L I V S L V S M F V L C W

812 TTCCGCTCCCTTTGCAACGCCTCGCGCCGCCAGAAGAGGAAGTCCCTTCACCTCATCGTCTCCCTGGTGTCCATGTTTGTGCTCTGCTGG

275 A P Y N S F Q L A E S L K K L G V I S G G C Q F G R T V D I

902 GCACCCTACAACTCCTTCCAATTGGCCGAAAGCCTGAAGAAGCTGGGCGTGATTAGTGGAGGCTGCCAGTTTGGCCGCACGGTGGACATC

305 G I L V S E S M G L S H C A L N P L L Y C F V G V K F R R E

992 GGGATCCTGGTGTCTGAGAGCATGGGCCTGTCACACTGTGCCCTGAACCCGCTGCTGTACTGCTTTGTGGGGGTGAAGTTTAGGAGGGAG

335 L T R M C K G L L G Q R F Y P G M K E W G G Q R K T R R P T

1082 CTGACCAGAATGTGCAAGGGGCTGCTGGGACAGAGGTTCTATCCAGGGATGAAGGAATGGGGAGGACAGAGGAAAACACGGAGGCCCACT

365 R S F S S A E S E N T H S I M A

1172 AGGTCCTTCAGCTCAGCAGAGAGTGAGAACACCCACTCTATCATGGCGTGAGCCAGCCACGTGCACAGATAGGGGTCTACCTGTGCCCGA

1262 GCACCTTTTTGTTGTTGTTCTGAACCCACAAGTCATCGTAAAGTCGTCAAGTTCACCACCCCCAACCCCCCATCCCATTGGTCTGCCCTG

1352 TACAGAGCTTGCGTGTGCCTGGTATCCAAACATGCATGGCTTGAAATGCAGTCACCGGTCGAGTCTGTGTAGCAAGCTACCTAGTTTAAA

1442 TATCAACAGTACTGCCACAGCAGCATAACATTTGATCTACATCATCATCAATATTTGGTTTGGCCTACATAGCCTTCGAGCAGCAGAGAG

1532 GCAGAAGACATAGAGAGGAGCGGCTGCATTCAGGCTCTACAACCCTCTGACCCATCTCAATCCCTTCATCAGTCAGGTGTTGCACATGTG

1622 TGTCAGGGCAGCAGCAACACAGGTAGTCTAGACTTTAGCTAACCACATATACTAAAATATCCACACAAGCCACCAGCCAACCAACCATAT

1712 ACTTACCATAGCATGAGTTAGAAGAGTATGAATATTATAGTATATTATGAAGTTATG**ATTTA**AGAGCATTGAAGAGAATCACAAAAAATG

1802 CTAGTTAACTATCAG**ATTTA**CATCAATCATCAACACAATGATCAGCTGATAGCCTATCCTCTTTACCCGATGTCAGTCATGTTTAGGAGG

1892 CACTTTAAATATATAGGTTTATGTTTTTGTTTTTTTAGGTAGTATGTTATAC**ATTTA**GAGATATCATTTTTTTAAAGGTGGTTGTCACTA

1982 GTTACCACAGGCACAAAGTCCAAATTGGCTATAATGTAAAAATTCATGAAAACAAATATTAGCTTTTAGGTCTTA**ATTTA**AGGTTAGGGT

2072 TAGGCATAAGGTTAGCAGTGTGGTTAGGGGTAAGGTTAGGTTTAAAATCAGATTTGAAGAAGAGAAATTGTAGAAATAGGCAGGAGTCTA

2162 GCCATAATTATGACTTTGTGGCTCTGGTAACTAGTGAGGACCGTGAAAGGTCTGTGCATCCCGGCCCAGGTGAGAGCCTGGAGAGAGGGA

2252 GAAAGAGAGAGAGGGAGCGGGGGATGTGGAGACTGACAGAAAAAGAGAGAGGAAGAGTGAATACAAGTCAAAAGGGGGACAGGTAATATT

2342 GGACTGTTGCTGAAAAGAGGAAACACAAATTGTTTATTGTGAAAATGACAAAATGGGAAAGAAATACAGATGTGCACATTGTGTCAAAGT

2432 GAGGAAAAAATGTATGTTATTTTGTTGTGATTCGTTCCTCTGGTGCTTCCTGATAGTGGCCCTGCTCCTACTGTAATAGGTCAAAATTAT

2522 GGTGCTTTTAAAGACAACACATTCTCAGGGAGGACATTTTTCATATAGTAGCCTATTTTCACTCTCTTCTTGTAATTTTGGTTAATGAAA

2612 TTCTACTGTCCATCCAATCGTATAGCCTTCCAAGTTAAAACCCCATCTGCCTCTCGTGTGACCATGTTATCCCATCTGCCTCTAGTGGTG

2702 TGACCATGTTAACCCCATCTGCCTCTAGTGTGAGCAGCAAAGACTGCATAATATGAAGGTGAGCAGTGAACAATTTCTCAAGCTGTTTGG

2792 TTTCCTGATAAGTTGATGACTATAATTTCAAGGAACTGGATAATTTCTTGCAAAATATATATTTTTAAATGTATGAAACCTACTTCCTCA

2882 AAAGGGCCAAGTCATCACTATATGTGTTACTCATATGTTGCCTTGTGTTTTTGAAGGCAGCAAACACTGATACCTTTCAAATCCTGCAGC

2972 AATAGTTTTTTCTTTGTTTGATTGCTTCATTTTTTCACTTGTTTTAGAGGGGAAACAAAGGTGTTAAGTAAGTCATGACAGACATGCAGC

3062 TTTACTTATGGCAAATGTCCACGTGTCCTTTGGTACCAGACTGTTATTCAACATTTTCTAATTCACTTCTTTAGATGAGATTGTTATACA

3152 TCAGTCAGCACAACACATATGTACATAGGATTGTATACATGTATTTCTGTGTTGTAGAAATCTTAGGAAGTAGAATAAAACATTGGGGGA

3242 AATGTACTTTTGATATATCAATAAATAAATGATATGCAAATATCGTAAAAAAAAAAAAAAAAAAAAA

**Fig. S3. Nucleotide and deduced amino acid sequences of rainbow trout CXCR3b cDNA (**EMBL accession number AJ888878)**.** The start and stop codons for translation, an in-frame stop codon in the 5’-UTR, and a polyadenylation signal in the 3’-UTR are highlighted in red. Potential *N*-glycosylation sites are in bold and underlined. Putative mRNA instability motifs (ATTTA) are in bold and boxed. The seven transmembrane domains are highlighted in green.
